# Novel hemostatic technique using a silicone gel dressing for tangential excision in burn surgery

**DOI:** 10.1186/cc13279

**Published:** 2014-03-17

**Authors:** A Osuka, Y Kuroki, H Kojima, M Sekido, S Okuma, S Onishi, M Ueyama

**Affiliations:** 1 Social Insurance Chukyo Hospital, Nagoya, Japan

## Introduction

The purpose of this study is to demonstrate the efficacy of our novel hemostatic technique for burn surgery. Significant bleeding remains a challenge in tangential excision of the burn wound. Although various techniques to reduce intraoperative blood loss have been described, there is an absence of uniformity and consistency in their application. In the literature, the blood loss in burn surgery is estimated to be at least 123 ± 106 ml per percentage body surface area excised [1]. Recently we developed a novel hemostatic technique using a silicone gel dressing (SI-AID^®^; ALCARE Co., Ltd, Tokyo, Japan) to stop intraoperative bleeding. Briefly, soon after tangential excision with the Humby knife, the wounds were sprayed with thrombin and with 1:100,000 adrenalin solution and wrapped tightly with SI-AID^® ^for a full 10 minutes. Burn wounds on limbs were tangentially excised under tourniquet control and wrapped with SI-AID^® ^before deflation of the tourniquet. After deflation of the tourniquets, we waited for a full 10 minutes. When the SI-AID^® ^was removed, any major bleeders were cauterized, and the grafts were applied after rinsing the wounds with warm saline.

## Methods

This is a prospective observational study. From 1 January to 31 October 2013 we collected preoperative and 24-hour postoperative hemoglobin levels from the patients who underwent tangential excision for burn injury, and calculated blood loss in the perioperative period. The data for amounts of blood transfusion, excised area, and harvest area were also collected.

## Results

Nine patients, 13 operations were included. The mean excised area was 8.3 ± 4.6% body surface area. Estimated blood loss was 35.3 ± 38.3 ml per percentage body surface area excised. Intraoperative transfusion requirements were 86.2 ± 134.5 ml per case. The mean skin graft take rate was 4.5 ± 2.2%.

## Conclusion

The application of our new technique during burn excision and grafting resulted in a remarkable reduction in blood loss and transfusion requirements.
